# Supplementary material for: Dopaminergic Degeneration Differentially Modulates Primary Motor Cortex Activity and Motor Behavior in Hemiparkinsonian Rats
Source: Brain Sci. 2025 Oct 18;15(10):1123. doi: 10.3390/brainsci15101123 (PMC12562322; doi:10.3390/brainsci15101123)
Supplement: Supplementary file 1 [file brainsci-15-01123-s001.zip › brainsci-3898071-supplementary.pdf]

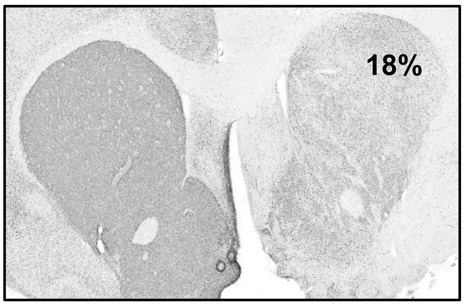

Supplementary Figure S1. Histological assessment of dopaminergic lesion of R13. Densitometric analysis of TH+ fibers in the striatum of R13 revealed 18 % loss of dopaminergic innervation from the SNc.

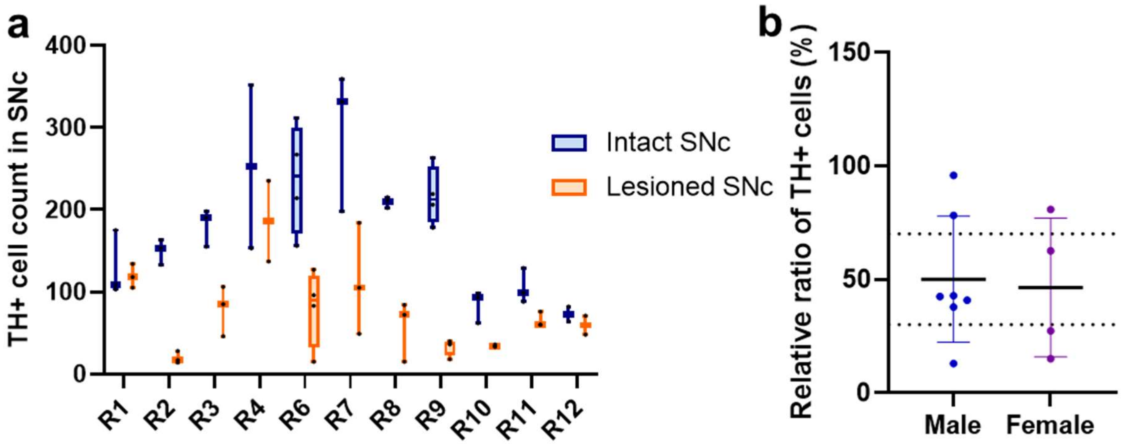

Supplementary Figure S2. Individual and sex-based analysis of dopaminergic lesion. (a) Histological quantification of TH+ neurons in each rat's intact and lesioned SNc. Data points represent the midbrain sections containing intact and lesioned SNc. Boxes represent the mean and SD, with min and max values. **(b)** Mann-Whitney analysis of the SNc lesion with sex as an independent variable ($U = 13$, $p = 0.927$).

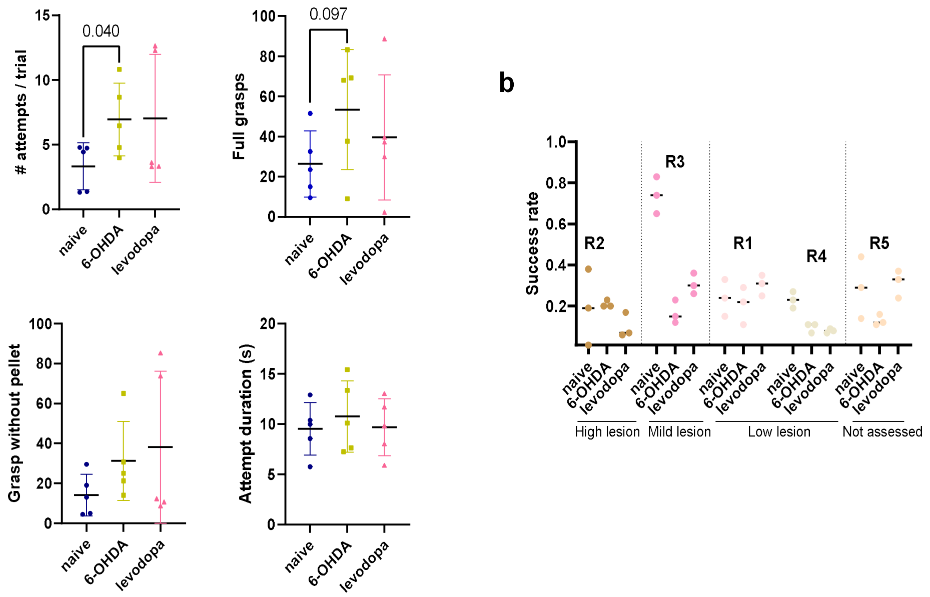

Supplementary Figure S3. Motor function assessment of rats 1 – 5 in the Single Pellet Reaching Test (SPRT). Rats 1 through 5 received 2 sessions of 15 trials each during the naive state, and 3 or 4 sessions of 15 trials each during the 6-OHDA lesioned state and the levodopa treatment state. Since insufficient baseline training occurred, data from these animals are analyzed separately from the main group with mixed effects linear regression models, including a fixed effect for state and a random effect for rat. Data are represented as mean ± SD. N = 5.

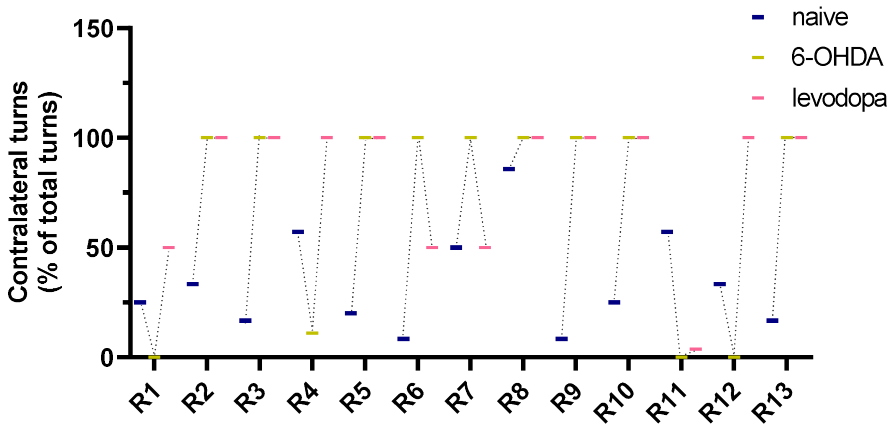

Supplementary Figure S4. Rotation preference. Ipsilateral and contralateral turns were counted for 15 min after apomorphine injection (s.c.) at the end of the SPRT in each state (naïve, 6-OHDA-lesioned, and levodopa-treated). Data are expressed as percentage of contralateral turns relative to ipsilateral turns to demonstrate rotation preference in each state.

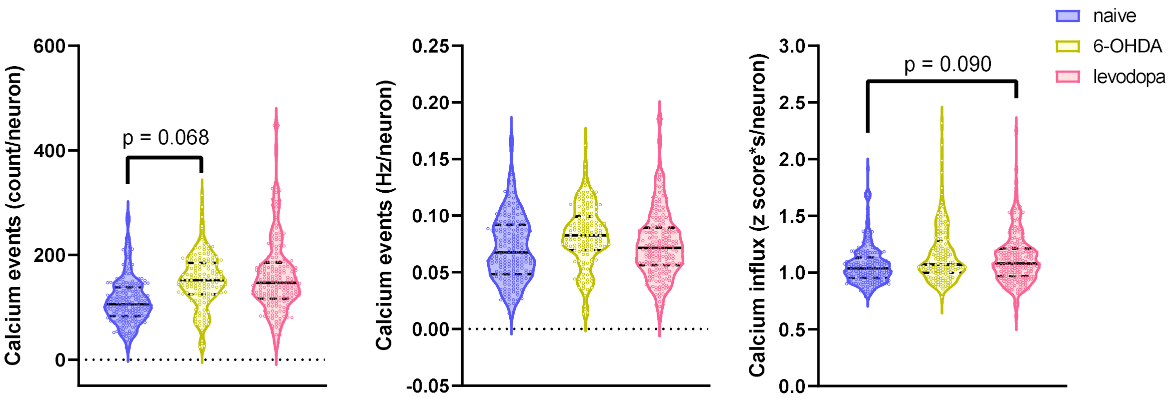

Supplementary Figure S5. Calcium activity in the primary motor cortex of rats. Alternative representation of M1 neuronal calcium activity of rats transitioning from a naïve, to a 6-OHDA-lesioned, to a levodopa-treated state. Data were analyzed using a mixed effects linear regression models with a fixed effect for state and a random effect for rat. Each dot represents a recorded neuron. Median and interquartile range are represented by filled and dotted lines respectively. The shape of the violin plots represents the distribution of individual data points. N = 91 neurons – naïve; N = 81 neurons – 6-OHDA; N = 90 neurons – levodopa.

Supplementary Table S1. Behavioral outcome comparisons of rats 1 through 5 in the SPRT.

	naïve vs. 6-OHDA		naïve vs. Levodopa		6-OHDA vs. Levodopa	
Variable	Mean difference (95% CI)	P-value	Mean difference (95% CI)	P-value	Mean difference (95% CI)	P-value
Attempts/trial (Performance)	3.62 (0.28, 6.96)	0.040	3.71 (-2.85, 10.26)	0.190	0.08 (-6.07, 6.23)	0.970
Full Grasps	27.07 (-7.77, 61.91)	0.097	26.40 (-4.59, 57.39)	0.400	-18.87 (-37.60, 9.87)	0.180
Grasp without pellet	17.00 (-7.36, 41.36)	0.120	23.93 (-25.01, 72.88)	0.250	6.93 (-39.86, 53.73)	0.700
Attempt Duration	1.23 (-3.44, 5.89)	0.510	0.17 (-2.97, 3.31)	0.890	-1.06 (-4.49, 2.37)	0.440

CI=confidence interval. Mean differences, 95% CIs, and p-values result from mixed effects linear regression models, including a fixed effect for state and a random effect for rat. For naïve vs. 6-OHDA comparisons, mean differences are interpreted as the difference in the mean outcome level for 6-OHDA minus naïve. For naïve vs. Levodopa comparisons, mean differences are interpreted as the difference in the mean outcome level for Levodopa minus naïve. For 6-OHDA vs. Levodopa comparisons, mean differences are interpreted as the difference in the mean outcome level for Levodopa minus 6-OHDA.

Supplementary Table S2. Total number of neurons recorded and analyzed in each rat during each treatment state.

Rat ID	State	Total neurons recorded
**R1**	Naïve	76
	6-OHDA	57
	Levodopa	82
**R2**	Naïve	66
	6-OHDA	38
	Levodopa	48
**R3**	Naïve	205
	6-OHDA	135
	Levodopa	205
**R4**	Naïve	60
	6-OHDA	116
	Levodopa	56
**R6**	Naïve	33
	6-OHDA	40
	Levodopa	13
**R7**	Naïve	146
	6-OHDA	149
	Levodopa	149
**R8**	Naïve	57
	6-OHDA	38
	Levodopa	96
